# Performance and optimization study of selected 4-terminal tandem solar cells

**DOI:** 10.1038/s41598-024-62085-0

**Published:** 2024-05-20

**Authors:** Zeinab shokrollahi, Mina Piralaee, Asghar Asgari

**Affiliations:** 1 Faculty of Physics, University of Tabriz, Tabriz, Iran; 2 Photonic Devices Research Group, Research Institute for Applied Physics and Astronomy, University of Tabriz, Tabriz, Iran; 3 School of Electrical, Electronic and Computer Engineering, University of Western Australia, Crawley, WA 6009 Australia

**Keywords:** Tandem solar cell, 4terminal tandem, Efficiency, Organic material, Perovskite, SCAPS, Photovoltaics, Solar cells

## Abstract

Tandem solar cells owing to their layered structure in which each sub-cell utilizes a certain part of the solar spectrum with reduced thermal losses, are promising applicants to promote the power conversion efficiency beyond the Shockley–Queisser limit of single-junction solar cells. This study delves into the performance and optimization of 4-terminal organic/silicon tandem solar cells through numerical simulations using SCAPS-1D software. The tandem architecture combining organic, perovskite, and silicon materials, shows potential in enhancing light absorption across the solar spectrum with complementary absorption spectra. Through innovative material exploration, optimization techniques are explored to advance the performance boundaries of organic/silicon tandem solar cells. The study employs the Beer–Lambert law to assess the impact of varied physical parameters on tandem solar cell efficiency, aiming to propose optimal configurations. Results indicate a maximum efficiency of 25.86% with P_3_HT:PC_70_BM organic active layer (150 nm thickness) and 36.8% with Cs_2_AgBi_0.75_Sb_0.25_Br_6_ active layer (400 nm thickness) in the studied 4-terminal tandem structures. These findings offer valuable insights into the complex physics of these tandem solar cells, for developing high-performance and commercially practical photovoltaic devices.

## Introduction

Despite their advantageous properties such as ease of processing, affordability, flexibility, and lightweight nature, organic solar cells demonstrate significantly lower efficiency compared to silicon counterparts due to their limited electron and hole mobility as well as constrained absorption coefficients^1–3^. Improving light absorption in organic solar cells can achieved through the design of materials with broad absorption spectra or the incorporation of absorbers with varying band gaps. Recent endeavors to enhance the power conversion efficiency (PCE) of organic solar cells encompass tactics like the development of high-performance donor and acceptor materials, integration of ternary active layers, adoption of tandem device architectures, and refinement of carrier transport layers and fabrication techniques^4–17^. Accordingly, there has been a notable enhancement in PCEs, exceeding 16% for single-junction cells and surpassing 17% for ternary and tandem configurations^18–22^.

Typically, solar radiation distribution reveals that approximately 47% falls within the infrared spectrum, 46% within the visible range, and merely 7% in the ultraviolet spectrum. The ultraviolet radiation because of higher frequency has a high probability of being scattered by the solar cell surface. On the other hand, infrared radiation, because of the long wavelength, has little chance of being absorbed inside the cell. Strategies to improve light absorption include designing multi-layer structures connected by narrower strips at bottom junctions(tandem) and applying mixtures of organic and inorganic materials(ternary) to capture the infrared spectrum efficiently.^23^. Typically, in tandem solar cells, the top cell features a wider band gap to capture high-energy photons, while the bottom cell, with a narrower band gap, absorbs the lower-energy photons transmitted from the top cell^24^.

Tandem solar cells have become a focal point due to their promise of achieving remarkable efficiency using cost-effective materials and processes. In the 4-terminal (4-T) tandem configuration, wide-bandgap and narrow-bandgap sub-cells are stacked mechanically. Conversely, the 2-terminal (2-T) tandem design necessitates fabricating sub-cells directly on top of one another, linked by a tunnel recombination junction.^25,26^.

Silicon and perovskite materials are viable choices for the bottom cell in tandem structures. Silicon has a proven record of accomplishment of efficiency in optoelectronic devices, while perovskite materials offer tunable band gaps and notable optoelectronic properties. The integration of perovskite and crystalline silicon technologies in tandems holds promise due to their potential for reduced manufacturing costs^27–38^.

The aim of this investigation is to assess the efficacy of 4-terminal tandem configurations employing the organic active layer P3HT:PCBM. Enhancing solar cell efficiency, the hole transport layer (PEDOT:PSS) and electron transport layer (PDINO) facilitate electron transfer and ensure optimal surface contact between the electrode and active layer^39,40^. To compute the optical and electronic characteristics of tandem solar cell devices integrating perovskite and crystalline silicon, Solar Cell Capacitance Simulator (SCAPS) utilized.

In the mechanically stacked 4-T tandem device, distinct values of short-circuit current density (Jsc), open-circuit voltage (Voc), and fill factor (FF) are assigned to the top and bottom cells, allowing for independent optimization of each sub-cell without requiring current matching conditions. The overall efficiency is calculated as the sum of the individual PCEs of the top and bottom cells^41,42^.

In general, in this paper, we have simulated 4-terminal tandem solar cells by replacing different layers (along with their accompanying transport layers), as top cell and bottom cell, in order to identify the optimal structure with the highest performance. The advantage of this method is to identify the optimal structure in terms of building layers and determine the physical and optical characteristics of the optimum structure. For the simplicity of calculations, by using the Beer-Lambert law, we have considered the spectrum passing from the top cell as the incident spectrum to the bottom cell.

Modeling.

**Figure 1:**
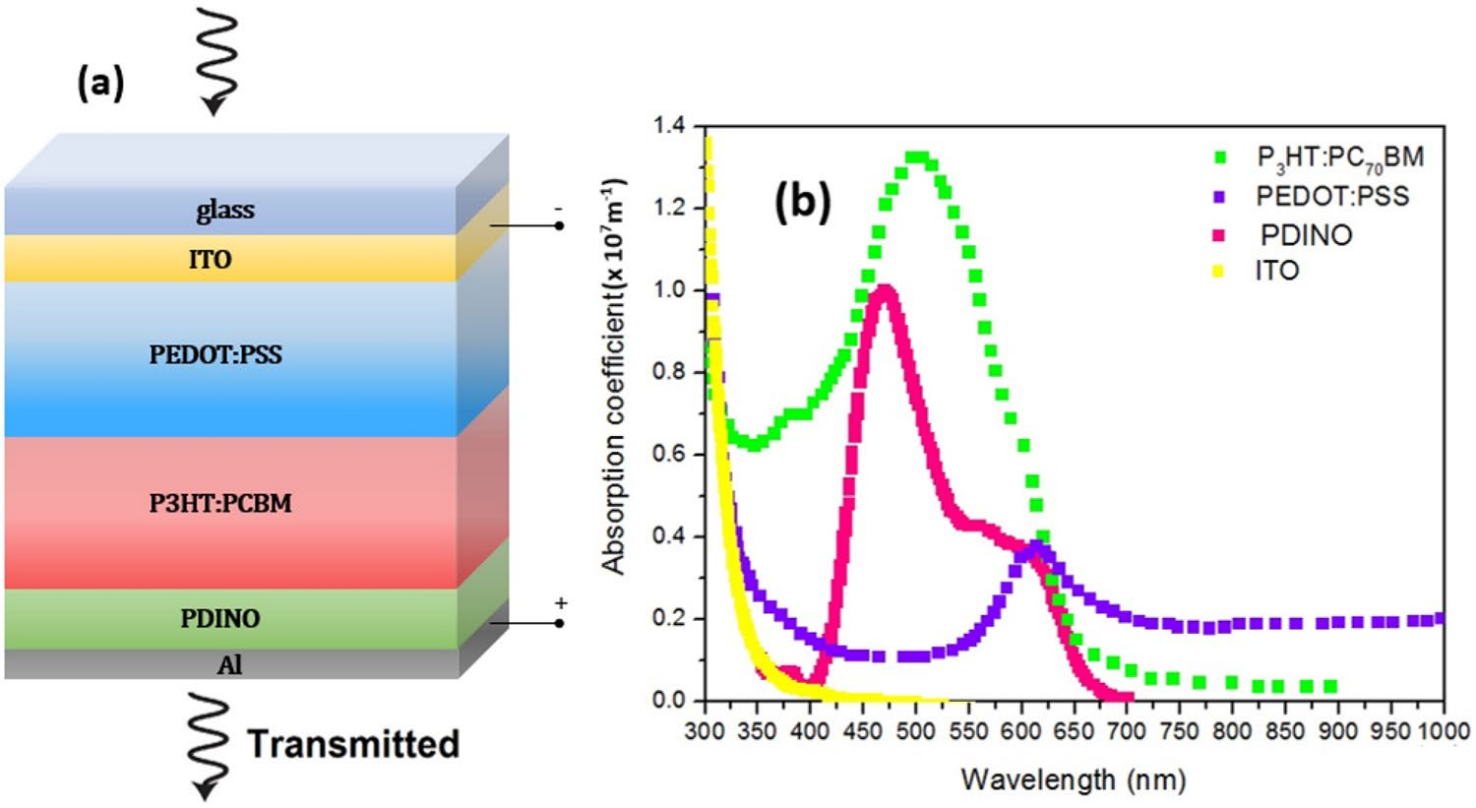
**(a)** Schematic of the top-cell of 4-terminal tandem structure with P_3_HT:PC_70_BM active layer, and (**b**) the absorption coefficient of the layers of the top-cell.

As the starting point, we consider two tandem structures with different top cells. A schematic of the layers used in the top cell in the first studied tandem structure with P_3_HT:PC_70_BM active layer shown in Fig. 1a and the absorption coefficient of the active layer is illustrated in Fig. 1b. All used parameters in the top-cells simulation have denoted in Table 1.

**Table 1:** The parameters used in each layer of the organic top-cell^46,47^.

Parameters	PEDOT:PSS(HTL)	P_3_HT:PC_70_BM	PDINO(ETL)
Thickness (μm)	30	100	5
${E}_{g} ({\text{eV}})$	1.5	1.5	2.98
*Χ* (eV)	3.4	3.9	4.1
${\varepsilon}_{r}$	3	3.8	5
${\mu }_{n} (\frac{{{\text{cm}}}^{2}}{\mathrm{V S}})$	$4.5\times {10}^{-3}$	$9\times {10}^{-3}$	$2\times {10}^{-6}$
${\mu }_{p} (\frac{{{\text{cm}}}^{2}}{\mathrm{V S}})$	$9.9\times {10}^{-5}$	$7\times {10}^{-3}$	${10}^{-3}$
${N}_{A} ({{\text{cm}}}^{-3})$	${10}^{21}$	8.17	0
${N}_{D} ({{\text{cm}}}^{-3})$	0	0	$2\times {10}^{21}$
${N}_{c} ({{\text{cm}}}^{-3})$	${10}^{22}$	${10}^{19}$	${10}^{19}$
${N}_{V} ({{\text{cm}}}^{-3})$	${10}^{22}$	${10}^{19}$	${10}^{19}$
${N}_{t} \left({{\text{cm}}}^{-3}\right)$	${10}^{9}$	$7.7\times {10}^{16}$	${10}^{9}$

The optical parameters (refractive index n and extraction coefficient k) of the functional layers are also could derived from absorption coefficients ($\alpha = \frac{(n + ik)\omega }{c}$)^43,44^. The light spectrum utilized for the top cell is the AM1.5 spectrum, while the spectrum transmitted through it is obtained using Beer–Lambert law, presented in Eq. (1):

1 $${\text{S}}\left(\uplambda \right)={{\text{S}}}_{0}\left(\uplambda \right)\times {\text{exp}}(\sum_{{\text{i}}=1}^{4}-({\mathrm{\alpha }}_{i}\left(\uplambda \right)\times {{\text{d}}}_{{\text{i}}})) $$

where, ${{\text{S}}}_{0}\left(\uplambda \right)$ is the primary solar spectrum, of AM 1.5, d is the thickness of the active layer and α is the absorption coefficient of the corresponding substance^45^. The top-cell of the organic structure has three main layers of PDINO/P_3_HT:PC_70_BM/PEDOT: PSS^46,47^. The main layer of this cell is the active layer of P_3_HT:PC_70_BM.

**Figure 2:**
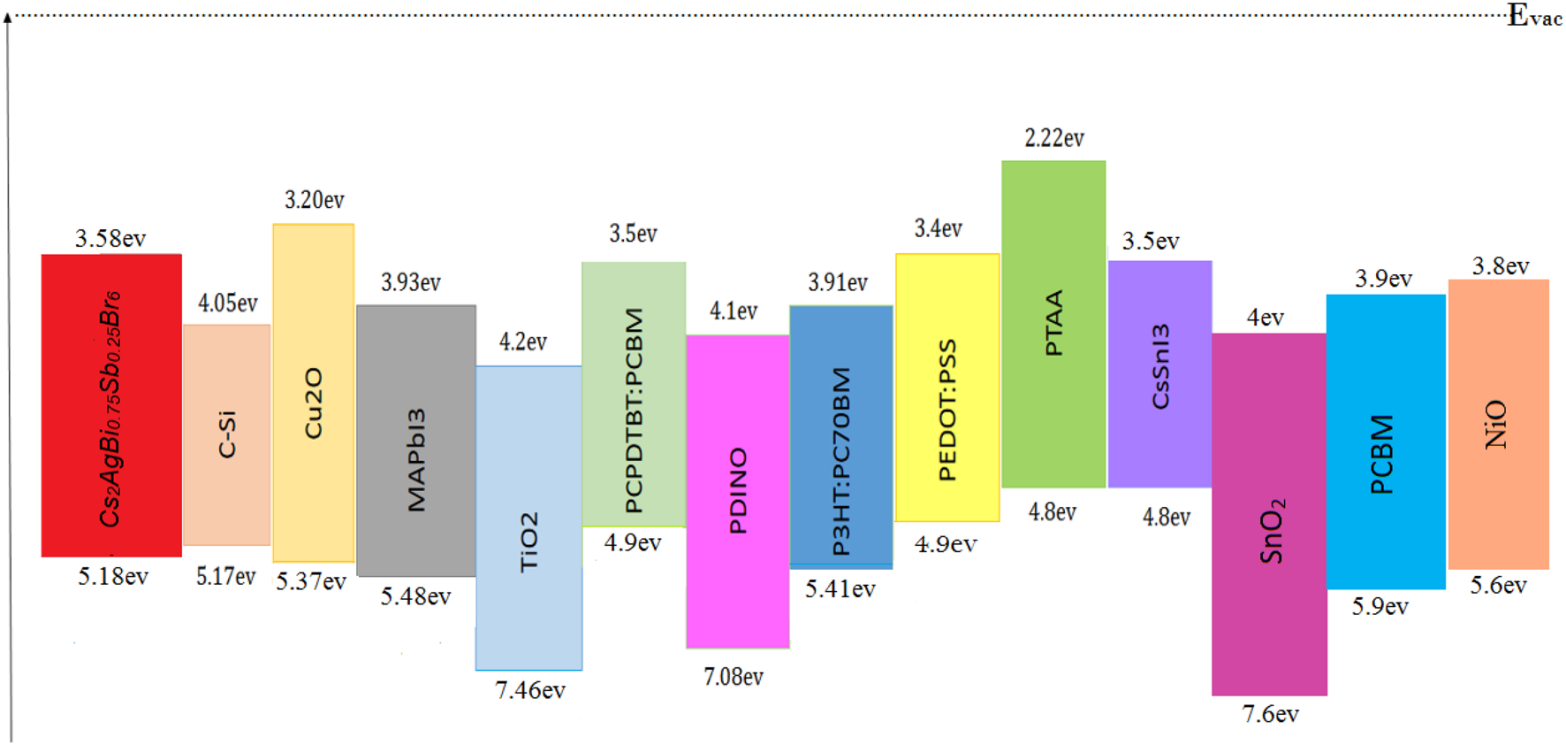
Diagram of the energy bands for materials used in this study.

The capture cross-section of electrons and holes trapping for two layers of ETL and HTL is about 9 × 10^–15^ cm^2^ and for the active layer P_3_HT:PC_70_BM it is about 1.5 × 10^–18^ cm^2^. The first and second surface defect density between the P_3_HT:PC_70_BM/PEDOT: PSS interface is 1.6 × 10^9^ cm^−3^ and 1.9 × 10^12^ cm^−3^, respectively. The capture cross-section of electrons and holes trapping for both are 10^–19^ cm^2^. The first and second surface defect densities between the P_3_HT:PC_70_BM/PDINO interface are 1.6 × 10^9^ cm^−3^ and 1.5 × 10^12^ cm^−3^, respectively, and the capture cross-section of electrons and holes trapping for both are 10^–19^ cm^2^. Figure 2 illustrates the energy band diagram of the materials employed in the structure, elucidating the distinctions among energy levels.

Using a parallel approach, we implemented the aforementioned methodology to analyze Cs_2_AgBi_0.75_Sb_0.25_Br_6_ perovskite solar cell as the top-cell. We computed the transmission spectrum within the perovskite sub-cell and subsequently utilized it as the input spectrum for the bottom-cell analysis.

**Figure 3:**
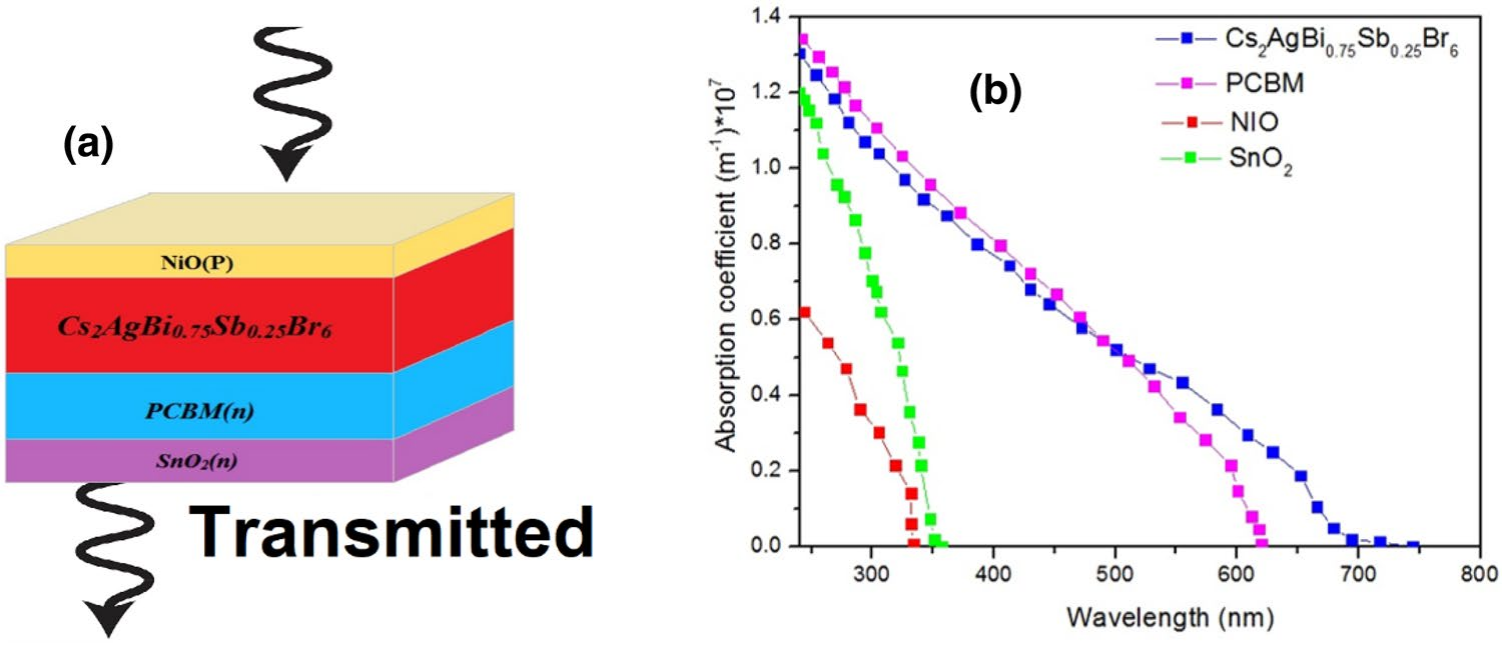
(**a**) Schematic of the top-cell of 4-terminal tandem structure with Cs_2_AgBi_0.75_Sb_0.25_Br_6_ perovskite active layer, and (**b**) the absorption coefficient of the layers of top-cell.

According to Fig. 3, it is evident that the material exhibits maximum absorption within the ultraviolet region of the solar spectrum, tapering off around 700 nm. Consequently, it can be inferred that the transmission spectrum of this cell surpasses that of the top-cell featuring the P_3_HT:PC_70_BM active layer, Hence, it is anticipated that the simulated structures will yield higher results for the bottom-cells, both in the single and tandem configurations. The used parameters for the simulation of the perovskite top-cell are included in Table 2**.**

**Table 2:** The used parameters for the simulation of the perovskite top-cell^46^.

	SnO_2_	PCBM	Cs_2_AgBi_0.75_Sb_0.25_Br_6_	NIO
Thickness (μm)	6	40	400	40
${E}_{g} ({\text{eV}})$	3.6	2	1.8	3.8
*Χ* (eV)	4	3.9	3.58	1.8
${\varepsilon}_{r}$	9	4	6.5	11.7
${\mu }_{n} (\frac{{{\text{cm}}}^{2}}{\mathrm{V S}})$	${10}^{2}$	${10}^{-2}$	$2$	${10}^{-3}$
${\mu }_{p} (\frac{{{\text{cm}}}^{2}}{\mathrm{V S}})$	25	${10}^{-2}$	$2$	${10}^{-3}$
${N}_{A} ({{\text{cm}}}^{-3})$	0	$0$	${10}^{13}$	0
${N}_{D} ({{\text{cm}}}^{-3})$	${5\times 10}^{14}$	${5\times 10}^{14}$	${10}^{17}$	$5\times {10}^{17}$
${N}_{c} ({{\text{cm}}}^{-3})$	${2.2\times 10}^{18}$	${10}^{21}$	${2.2\times 10}^{18}$	$2.5\times {10}^{20}$
${N}_{V} ({{\text{cm}}}^{-3})$	${1.8\times 10}^{19}$	$2\times {10}^{20}$	${1.8\times 10}^{19}$	$2.5\times {10}^{20}$
${N}_{t} \left({{\text{cm}}}^{-3}\right)$	–	–	${10}^{14}$	–
Capture cross section electrons (${{\text{cm}}}^{2}$)	–	–	${10}^{-15}$	–
Capture cross section holes (${{\text{cm}}}^{2}$)	–	–	${10}^{-15}$	–

## Results and discussion

**Figure 4:**
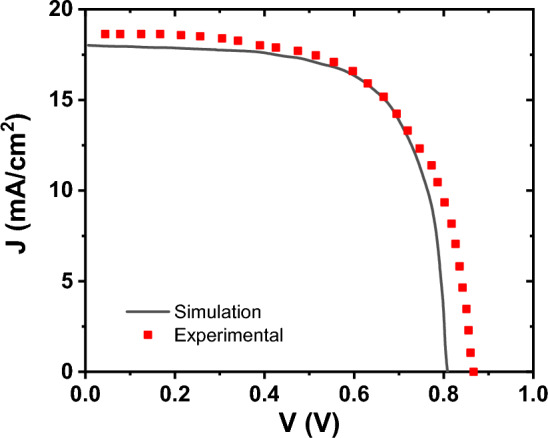
J–V curve of the PDINO/P_3_HT:PC_70_BM/PEDOT: PSS top-cell for thickness of 200 nm^46^.

Utilizing the absorption data and parameters outlined in Table 1, for each layer, we conducted simulations on the top-cell, with results for three varying thicknesses of the active layer documented in Table 3. To verify the precision of our findings, a comparison between experimental and simulation results for the cells with thickness of 200 nm is depicted in Fig. 4. which shows a good agreement between our model and experimental.

**Table 3:** The performance parameters of the top-cell *PDINO/P*_*3*_*HT:PC*_*70*_*BM/PEDOT: PSS* under AM1.5 for different thicknesses.

PDINO/P_3_HT:PC_70_BM/PEDOT:PSS	${V}_{OC}$	${J}_{SC}$	${\text{FF}}$	PCE
Thickness = 100 nm	0.80	12.97	80.32	8.38
Thickness = 150 nm	0.80	15.86	74.69	9.57
Thickness = 200 nm	0.80	18	69.57	10.14
Experimental^44^	0.84	18.64	64.6	10.19

**Figure 5:**
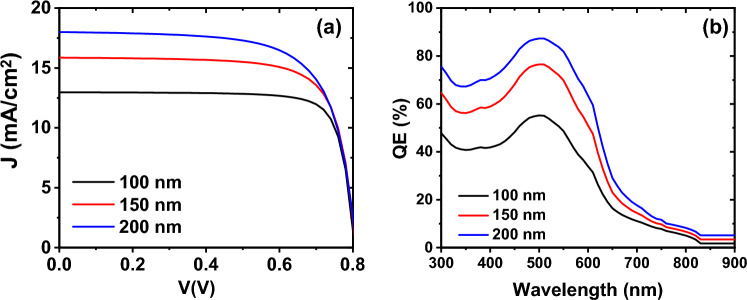
**(a)** The current–voltage, (**b**) external quantum efficiency of top-cell with three different thicknesses of P_3_HT:PC_70_BM active layer.

As depicted in Table 3, increasing the thickness of the top cell correlates with enhanced efficiency and current density of the cell. Nonetheless, due to loss mechanisms and carrier recombination, the transport of carriers becomes constrained, thereby leading to a decrease in the filling factor. This phenomenon is well illustrated by the current–voltage and quantum efficiency plots in Fig. 5.

**Figure 6:**
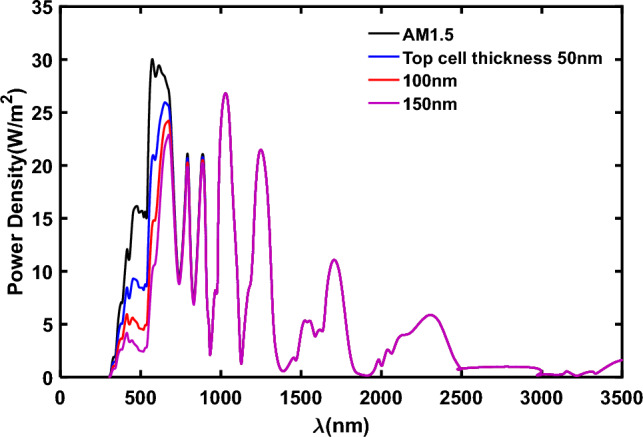
The transmission spectrum of the top-cell in three different thicknesses of P_3_HT:PC_70_BM.

**Figure 7:**
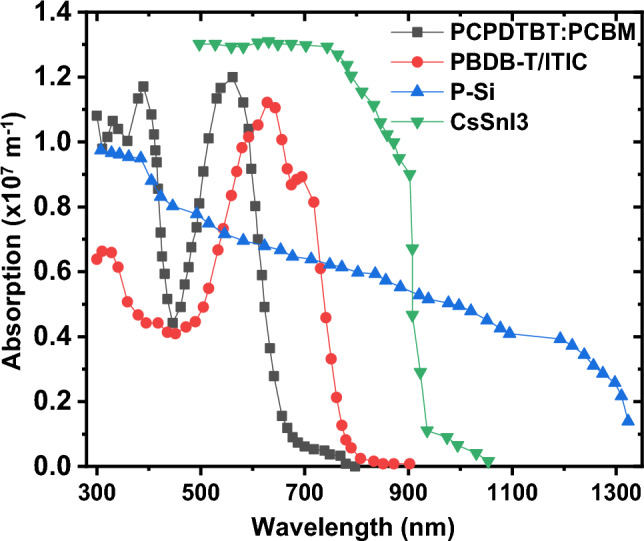
The absorption coefficient of the simulated bottom cells.

The transmission spectrum of the cell has been acquired across three different thicknesses: 50 nm, 100 nm, and 150 nm, as depicted in Fig. 6. Figure 6 demonstrates that as the active layer thickness increases, the cell absorbs more light, resulting in decreased light transmission through the cell. Consequently, the bottom cell exhibits reduced efficiency at higher thicknesses of the top cell. Subsequently, utilizing the parameters from Tables 4 and 5 for each bottom cell layer and the corresponding absorption data depicted in Fig. 7, we conducted simulations for four types of bottom cells: P-Si, PBDB-T: ITIC, PCPDTBT: PCBM, and CsSnI_3_. These simulations utilized the incident light spectrum filtered by the top cell with a thickness of 150 nm as the input for the bottom cells.

**Table 4:** The used parameters for each layer in the bottom-cells simulation^47–52^.

Parameters	P + Si	P Si	n + Si	PPTA	CsSnI_3_	TiO_2_	PEDOT:PSS
Thickness (μm)	20	80	0.5	0.15	0.70	0.14	0.03
${E}_{g} ({\text{eV}})$	1.12	1.12	1.12	2.58	1.3	3.26	2.9
*Χ* (eV)	4.05	4.05	4.05	2.22	3.5	4.2	2.2
${\varepsilon}_{r}$	11.9	11.9	11.9	2.9	9.93	10	3
${\mu }_{n}(\frac{{{\text{cm}}}^{2}}{\mathrm{V S}})$	$1.4\times {10}^{3}$	$1.4\times {10}^{3}$	$1.4\times {10}^{3}$	$2\times {10}^{-3}$	$1.5\times {10}^{3}$	$1\times {10}^{2}$	${10}^{-4}$
${\mu }_{p}(\frac{{{\text{cm}}}^{2}}{\mathrm{V S}})$	$4.5\times {10}^{2}$	$4.5\times {10}^{2}$	$4.5\times {10}^{2}$	$4\times {10}^{-3}$	$5.85\times {10}^{2}$	$2.5\times {10}^{1}$	${10}^{-3}$
${N}_{A}({{\text{cm}}}^{-3})$	$5\times {10}^{19}$	$1\times {10}^{16}$	0	$1\times {10}^{17}$	$1\times {10}^{15}$	0	$3\times {10}^{18}$
${N}_{D}({{\text{cm}}}^{-3})$	0	0	$1\times {10}^{20}$	0	0	$1\times {10}^{17}$	0
${N}_{c}({{\text{cm}}}^{-3})$	${2.819\times 10}^{19}$	${2.819\times 10}^{19}$	${2.819\times 10}^{19}$	${2.22\times 10}^{18}$	${10}^{18}$	${1.8\times 10}^{19}$	${1\times 10}^{22}$
${N}_{V}({{\text{cm}}}^{-3})$	${1.04\times 10}^{19}$	${2.6\times 10}^{19}$	${2.6\times 10}^{19}$	${1.8\times 10}^{19}$	${10}^{18}$	${3.5\times 10}^{19}$	${1\times 10}^{22}$
${N}_{t}\left({{\text{cm}}}^{-3}\right)$	${10}^{12}$	${10}^{12}$	${10}^{12}$	-	${10}^{18}$	-	${10}^{15}$
Capture cross section electrons(${{\text{cm}}}^{2}$)	${10}^{-14}$	${10}^{-14}$	${10}^{-14}$	-	${10}^{-15}$	-	${10}^{-9}$
Capture cross section holes(${{\text{cm}}}^{2}$)	${10}^{-14}$	${10}^{-14}$	${10}^{-14}$	-	${10}^{-15}$	-	${10}^{-10}$

**Table 5:** The used parameters for each layer in the bottom-cells simulation^47–52^.

*Parameter*	PCPDTBT:PCBM	TiO_2_	Cu_2_O	MAPbI_3_	TiO_2_	CuI	PBDB-T/ITIC	PFN-Br
Thickness (μm)	0.15	0.008	0.14	0.3	0.14	0.040	0.100	0.005
${E}_{g} ({\text{eV}})$	1.4	3.2	2.17	1.5	3.26	2.98	1.2	2.98
*Χ* (eV)	4.1	3.9	3.2	3.93	4.2	2.1	4.03	4
${\varepsilon}_{r}$	3	9	7.11	10	10	6.5	3.65	5
${\mu }_{n} (\frac{{{\text{cm}}}^{2}}{{\text{V}}.{\text{S}}})$	$6\times {10}^{-3}$	2 $\times {10}^{1}$	$2\times {10}^{2}$	$1$	${10}^{2}$	$1.69\times {10}^{-4}$	$3.1\times {10}^{-4}$	2 $\times {10}^{-6}$
${\mu }_{p }(\frac{{{\text{cm}}}^{2}}{{\text{V}}.{\text{S}}})$	${10}^{-4}$	${10}^{1}$	$8\times {10}^{1}$	$1$	$2.5\times {10}^{1}$	$1.69\times {10}^{-4}$	$3.2\times {10}^{-4}$	${10}^{-4}$
${N}_{A} ({{\text{cm}}}^{-3})$	$8\times {10}^{17}$	0	${10}^{18}$	${1 0}^{9}$	0	$2\times {10}^{18}$	0	0
${N}_{D} ({{\text{cm}}}^{-3})$	0	${10}^{17}$	0	${10}^{9}$	${10}^{17}$	0	0	$9\times {10}^{18}$
${N}_{c} ({{\text{cm}}}^{-3})$	${10}^{19}$	${10}^{21}$	${2.02\times 10}^{18}$	${2.75\times 10}^{18}$	${2\times 10}^{17}$	${10}^{22}$	${10}^{19}$	${10}^{19}$
${N}_{V} ({{\text{cm}}}^{-3})$	${10}^{19}$	${2\times 10}^{20}$	${1.1\times 10}^{19}$	${3.9\times 10}^{18}$	${6\times 10}^{17}$	${10}^{22}$	${10}^{19}$	${10}^{19}$
${N}_{t} \left({{\text{cm}}}^{-3}\right)$	${5\times 10}^{15}$	–	–	${10}^{15}$	–	${10}^{9}$	${10}^{12}$	${10}^{9}$
Capture cross section electrons (${{\text{cm}}}^{2}$)	1.5 × ${10}^{-18}$	–	–	${10}^{-15}$	–	${10}^{-15}$	9 × ${10}^{-15}$	${10}^{-15}$
Capture cross section holes(${{\text{cm}}}^{2}$)	1.5 × ${10}^{-18}$	–	–	${10}^{-15}$	–	${10}^{-15}$	9 × ${10}^{-15}$	${10}^{-15}$

**Figure 8:**
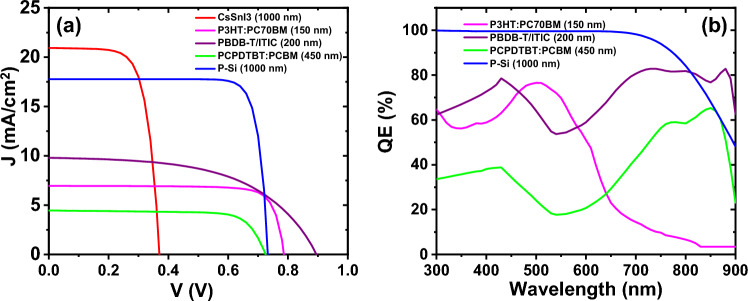
**(a)** The current–voltage, (**b**) quantum efficiency for simulated bottom cells.

Afterward, we computed the outcomes stemming from the simulation of the bottom cells within the tandem configuration, utilizing the filtered spectrum. The comprehensive efficiency of the 4-terminal tandem structure is detailed in Table 6. In the presented results, the efficiency of the bottom-cells was computed based on the spectrum filtered by the top-cell of 150 nm thickness. Subsequently, the total efficiency of the 4-terminal tandem structure was calculated for each cell. Among these cells, the 4-terminal tandem structure with the silicon solar cell bottom-cell exhibited the highest efficiency, reaching approximately 25.86%. In Fig. 8, we depict the external quantum efficiency and current–voltage density for all the cells listed in Table 6.

**Table 6:** The performance parameters of the bottom cells along with the efficiency of the 4-terminal tandem structure.

Bottom cells (filtered AM1.5G)	${V}_{OC}$	${J}_{SC}$	${\text{FF}}$	PCE	PCE (tandem)
P + Si/P Si (1000 nm)/n + Si	0.73	17.77	83.72	16.29	25.86
TiO_2_/CsSnI_3_ (1000 nm)/PTAA	0.36	21.82	68.61	8.23	17.80
CuI/PBDB-T:ITIC (200 nm)/PFN-Br	0.89	9.79	54.24	7.10	16.67
PEDOT:PSS/PCPDTBT:PCBM (450 nm)/TiO_2_	0.72	6.83	75.62	5.56	15.13

Based on the data presented in Fig. 8a, the current density of the bottom-cells varies significantly based on the material type and the optical properties, particularly the absorption coefficient of each layer. Among the results obtained, the most optimal performance is observed in the silicon cell, as indicated by its graph resembling the behavior of an ideal diode and consequently exhibiting higher efficiency than other cells. Furthermore, Fig. 8b illustrates that the external quantum efficiency of the silicon bottom cell attains the maximum value across the entire wavelength spectrum*.*

**Figure 9:**
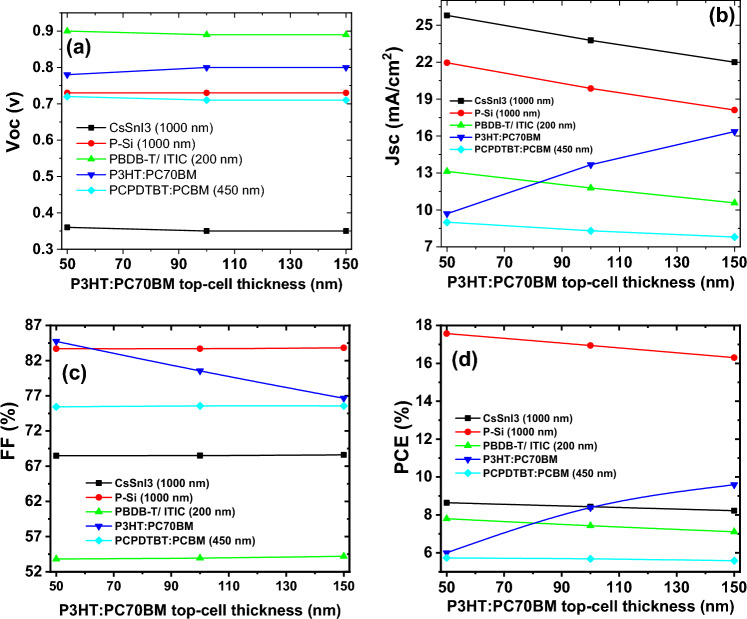
Comparison of the characteristic parameters of the top-cell and bottom-cells: (**a**) open circuit voltage, (**b**) short circuit current density, (**c**) filling factor, (**d**) power conversion efficiency as a function of top-cell thicknesses.

The performance of 4 T-tandem solar cells has evaluated across varying top-cell thicknesses. Figure 9 illustrates the characteristic parameters of bottom-cells concerning the thickness of the top cell, also the characteristic parameters of top-cell with variation of P3HT: PCBM /PEDOT: PSS thickness analyzed.

The open circuit voltage of the bottom cells, indicated by their respective thicknesses as depicted in the figures, and the top cell (PDINO/P3HT:PC70BM/PEDOT:PSS), are found to be independent of the thickness of the top cell. Figure 9a illustrates the variation of the open circuit voltage for both the bottom-cells and the top-cell concerning the thickness of the top cell.

According to Fig. 9b, the current density of the bottom cells decreases with an increase in the thickness of the top cell. This phenomenon attributed to a greater reduction in the transmitted light spectrum reaching the bottom cells, resulting in reduced absorption by the bottom cell. Notably, increasing the thickness of the top cell leads to an increase in the current density of the top cell (represented by the green line).

Moreover, as the top cell thickness increases, the filling factor, as shown in Fig. 9c, exhibits an almost constant behavior, while the top cell's fill factor demonstrates a decreasing trend due to an increased recombination rate.

**Figure 10:**
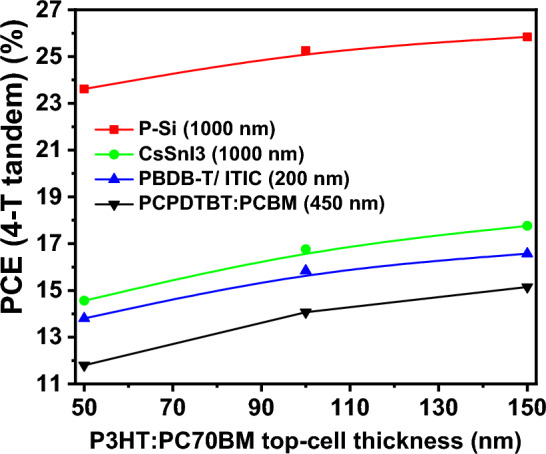
The variation of total PCE of the 4-terminal tandem structure with different bottom-cells as a function of top-cell thickness.

The outcomes of this investigation reveal a decreasing trend in the power conversion efficiency of the bottom cells and an increasing trend in that of the top cell, as depicted in Fig. 9d. Nevertheless, the total power conversion efficiency of the tandem structure experiences an increase with the thicknesses of the top cell, as illustrated in Fig. 10.

**Figure 11:**
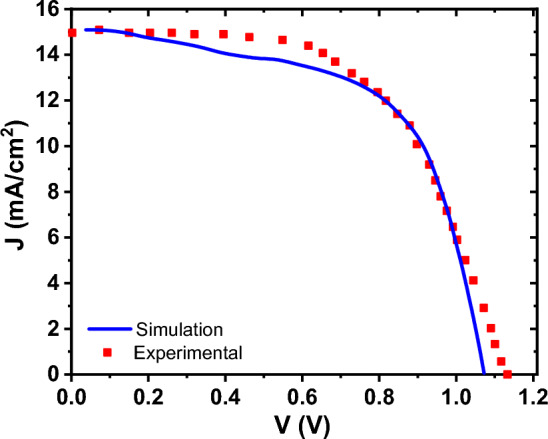
The current–voltage for the perovskite cell, comparison of our model with experimental results.

In order to verify the accuracy of our model concerning perovskite top-cells, we utilized a perovskite cell with a thickness of 400 nm, along with other crucial parameters drawn from Ref.^53^ and Table 2. The comprehensive comparison between experimental findings and simulation outcomes for the cell elaborated in Table 7 and illustrated in Fig. 11.

**Table 7:** The performance parameters for the perovskite cell considered in standalone conditions.

Top perovskite sub cell (1.8 eV)	${V}_{OC}$	${J}_{SC}$	${\text{FF}}$	PCE
Simulation	0.98	15.05	58.97	8.72
Experimental^51^	1.12	15.1	58	9.80

The findings presented in Table 7, coupled with the insights from Fig. 11, demonstrate a good agreement between the simulation and experimental data. Subsequently, we investigated the performance of the perovskite cell by varying the thickness of the perovskite layer, as outlined in Table 8. Our findings reveal that increasing the thickness of the perovskite layer maintains consistent effects on short-circuit current density and solar cell efficiency, leading to enhancements in both parameters while the fill factor (FF) and open-circuit voltage (V_OC_) remain practically unchanged. While augmenting the thickness of perovskite enhances the power conversion efficiency of the cells, it concurrently reduces the transmitted spectrum and diminishes the efficiency of bottom-cells.

**Table 8:** The performance parameters of the perovskite solar cell with different perovskite layer thicknesses.

NiO/Cs_2_AgBi_0.75_Sb_0.25_Br_6_/PCBM/SnO_2_	${V}_{OC}$	${J}_{SC}$	${\text{FF}}$	PCE
300 nm	1.37	14.08	74.42	14.41
400 nm	1.37	15.47	73.49	15.69
500 nm	1.38	16.36	72.86	16.48
600 nm	1.38	16.93	72.44	16.97
700 nm	1.38	17.27	72.16	17.25

**Figure 12:**
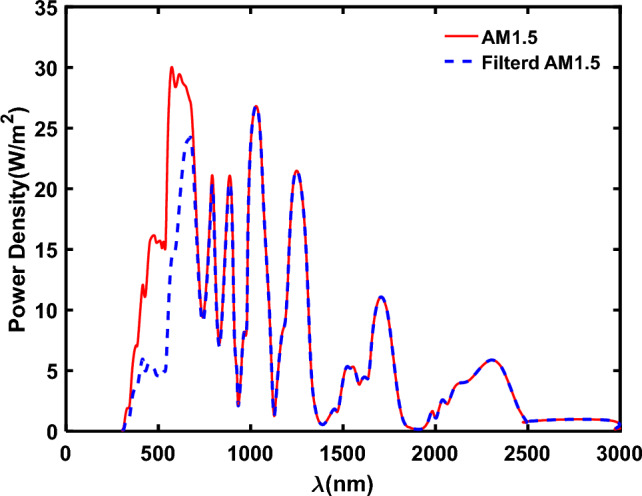
The filtered spectrum by the perovskite top-cell with 400 nm thickness in comparison with AM1.5 spectrum.

Figure 12 illustrates the transmission spectrum of perovskite solar cells functioning as the top-cell, featuring a 400 nm perovskite layer thickness. In the context of 4 T-tandem solar cells incorporating the perovskite top-cell, the transmitted spectrum serves as the input spectrum for the bottom-cells, with corresponding performance parameters outlined in Table 9.

**Table 9:** The performance parameters of the bottom-cells for the filtered spectrum passing through the 400 nm thickness of the top-cell.

Bottom cells (filtered AM1.5G)	${V}_{OC}$	${J}_{SC}$	${\text{FF}}$	PCE	PCE (tandem)
P + Si/P Si (20 μm)/n + Si	0.67	32.47	83.50	19.74	35.43
Cu_2_O/MAPbI_3_/TiO_2_	0.99	15.35	62.68	10.68	26.37
TiO_2_/CsSnI_3_ (1000 nm)/PTAA	0.37	36.26	66.29	9.68	25.37
PEDOT:PSS/PCPDTBT:PCBM (450 nm)/TiO_2_	0.71	11.65	74.72	6.67	22.36
PEDOT:PSS/P3HT:PC_70_BM (300 nm)/PDINO	0.79	12.36	62.56	6.58	22.27
CuI/PBDB-T:ITIC (200 nm)/PFN-Br	0.90	11.65	53.98	6.10	21.79

Based on the findings presented in Table 9, the silicon bottom-cell demonstrates the highest efficiency at approximately 19.74%. Consequently, the overall efficiency of the 4-terminal tandem structure reaches approximately 35.43%. Notably, comparative data indicates that the efficiency of two-terminal tandem solar cells with identical structures was approximately 24.4%^47^. In Table 10, we have juxtaposed the performance parameters of the silicon bottom-cell in standalone conditions with experimental outcomes from analogous studies. Notably, the simulation and experimental data exhibit a high degree of concordance^45^.

**Table 10:** Simulation (current work) and experimental photovoltaic parameters for the bottom silicon sub-cell considered in standalone condition with the parameters and thickness reported in the literature.

Bottom silicon sub-cell 1.2 eV	${V}_{OC}$	${J}_{SC}$	${\text{FF}}$	PCE
Our simulation	0.68	34.81	83.60	19.83
Experimental result^46^	0.62	37.7	78	18.9

**Figure 13:**
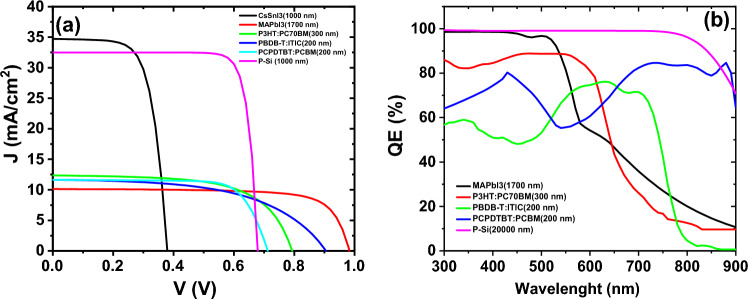
(**a**) The current–voltage, and (**b**) quantum efficiency for studied bottom- sub-cells in presence of peroveskite top-cell.

The peak efficiencies of MAPbI_3_ and CsSnI_3_ perovskites are 10.68% and 9.68%, respectively. These values contribute to the overall efficiency of the 4-terminal tandem, resulting in efficiencies of 26.37% and 25.37%, respectively. Please refer to Fig. 13 for the current–voltage characteristics and external quantum efficiency of all bottom cells.

## Conclusion

In this study, we investigated the efficiency of tandem solar cells, a key challenge being the optimization of structure absorption through tailored material selection for each sub-cell. Using SCAPS-1D, we analyzed the performance of 4-T tandem solar cells comprising perovskite and organic materials. Our findings indicate that properly configuring the parameters of each sub-cell can result in higher efficiency compared to 2-T counterparts. We calculated the transmission spectrum of the top cell to serve as input for the bottom cells, explored various thicknesses, and assessed overall efficiency. By employing different materials for top and bottom cells, we simulated results and compared them with experimental data. Our work contributes to enhancing the design of tandem structures, with the highest calculated efficiency reaching 25.86% for the top-cell with P3HT:PC70BM active layer and 35.43% for the top-cell with Cs_2_AgBi_0.75_Sb_0.25_Br6 active layer for the silicon bottom cell.

## Data Availability

The datasets used during the current study are available from the corresponding author on reasonable request.
